# Supplementary material for: The interactome of intact mitochondria by cross-linking mass spectrometry provides evidence for coexisting respiratory supercomplexes
Source: Mol Cell Proteomics. 2017 Dec 8;17(2):216–32. doi: 10.1074/mcp.RA117.000470 (PMC5795388; doi:10.1074/mcp.RA117.000470)
Supplement: Supplemental Data [file supp_RA117.000470_133922_0_supp_23978_fzffwf.zip › spectra_annotation/mito_DR_spectra_annotation/221-1-9-1-14-1.pdf]

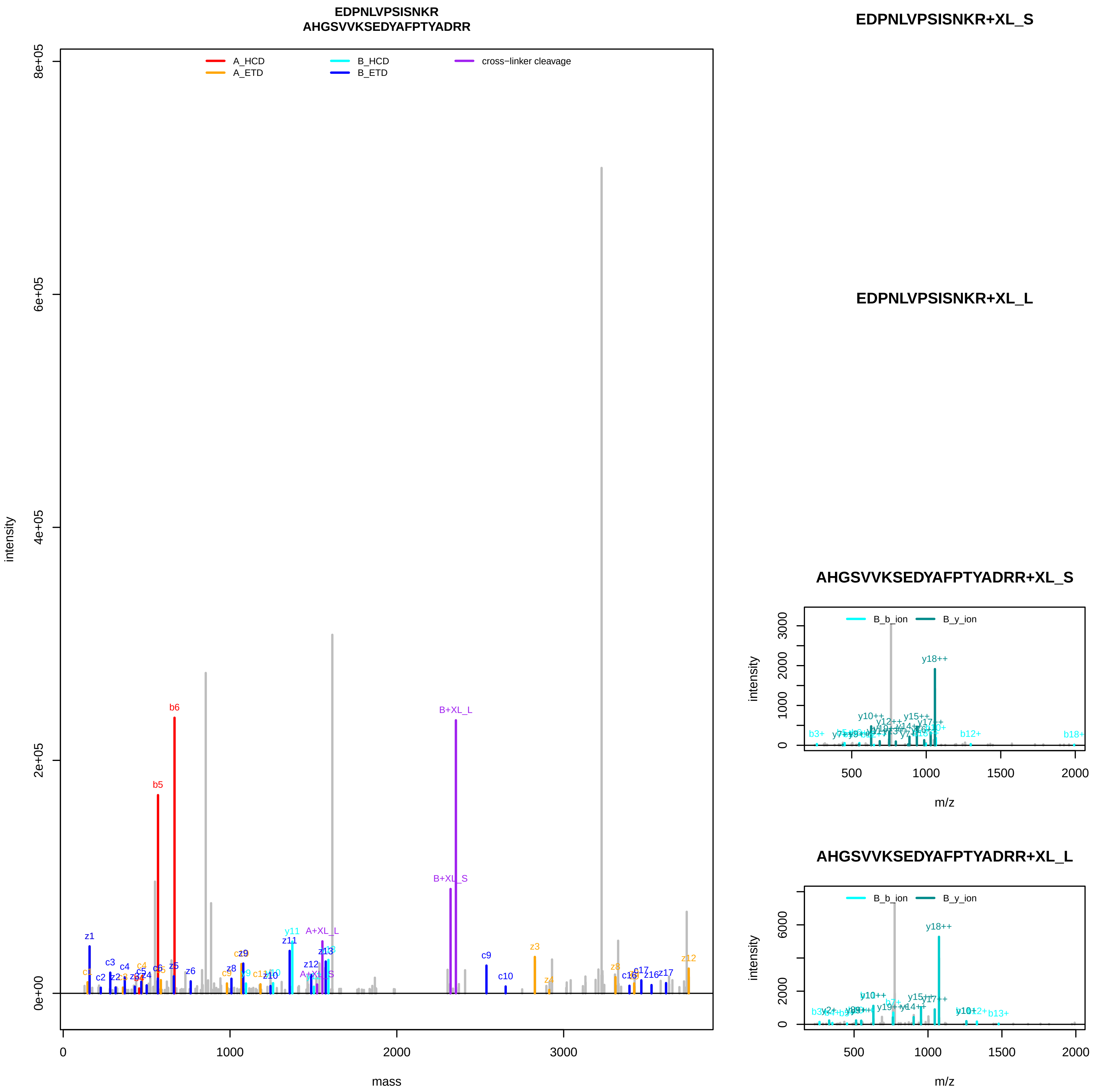
EDPNLVPSISNKR
AHGSVVKSEDYAFPTYADRR
A_HCD
A_ETD
B_HCD
B_ETD
cross−linker cleavage
intensity
mass
0e+00
2e+05
4e+05
6e+05
8e+05
0
1000
2000
3000
b5
b6
z1
c3
c4
c5
c6
z5
z6
z8
z9
z11
y11
A+XL_L
A+XL_S
z12
z13
B+XL_L
B+XL_S
c9
c10
z3
z4
z8
c16
c17
z16
z17
z12
EDPNLVPSISNKR+XL_S
EDPNLVPSISNKR+XL_L
AHGSVVKSEDYAFPTYADRR+XL_S
B_b_ion
B_y_ion
y18++
y10++
y15++
b3+
b12+
b18+
m/z
500
1000
1500
2000
0
1000
2000
3000
AHGSVVKSEDYAFPTYADRR+XL_L
B_b_ion
B_y_ion
y18++
y10++
y15++
y17++
b13+
0
2000
6000
